# Glioblastoma-derived cells *in vitro* unveil the spectrum of drug resistance capability – comparative study of tumour chemosensitivity in different culture systems

**DOI:** 10.1042/BSR20170058

**Published:** 2017-06-21

**Authors:** Monika Witusik-Perkowska, Magdalena Zakrzewska, Beata Sikorska, Wielislaw Papierz, Dariusz J. Jaskolski, Janusz Szemraj, Pawel P. Liberski

**Affiliations:** 1 Department of Medical Biochemistry, Medical University of Lodz, Mazowiecka 6/8, 92-215 Lodz, Poland; 2 Department of Molecular Pathology and Neuropathology, Medical University of Lodz, Czechoslowacka 8/10, 92-216 Lodz, Poland; 3 Department of Pathomorphology, Medical University of Lodz, Czechoslowacka 8/10, 92-216 Lodz, Poland; 4 Department of Neurosurgery and Oncology of Central Nervous System, Medical University of Lodz, Barlicki University Hospital, Kopcińskiego 22, Lodz 90-153, Poland

**Keywords:** apoptosis, autophagy, cancer drug resistance, cancer stem cells, cell culture, epithelial-to-mesenchymal transition, glioblastoma

## Abstract

Resistance to cancer drugs is a complex phenomenon which could be influenced by *in vitro* conditions. However, tumour-derived cell cultures are routinely used for studies related to mechanisms of drug responsiveness or the search for new therapeutic approaches. The purpose of our work was to identify the potential differences in drug resistance and response to treatment of glioblastoma with the use of three *in vitro* models: traditional adherent culture, serum-free spheroid culture and novel adherent serum-free culture.

The experimental models were evaluated according to ‘stemness state‘ and epithelial-to-mesenchymal transition (EMT) status, invasion capability and their expression pattern of genes related to the phenomenon of tumour drug resistance. Additionally, the response to drug treatments of three different culture models was compared with regard to the type of cell death.

Multi-gene expression profiling revealed differences between examined culture types with regard to the expression pattern of the selected genes. Functionally, the examined genes were related to drug resistance and metabolism, DNA damage and repair and cell cycle control, and included potential therapeutic targets.

Cytotoxicity analyses confirmed that environmental factors can influence not only the molecular background of glioblastoma drug-resistance and efficiency of treatment, but also the mechanisms/pathways of cell death, which was reflected by a distinct intensification of apoptosis and autophagy observed in particular culture models. Our results suggest that parallel exploitation of different *in vitro* experimental models can be used to reveal the spectrum of cancer cell resistance capability, especially regarding intra-heterogeneous glioblastomas.

## Introduction

Tumour-derived cell cultures are a common model used in studies of drug resistance mechanisms or the search for new therapeutic approaches. However, the development of a representative *ex vivo* model is fraught with problems, especially when examining highly heterogeneous tumours such as glioblastomas, as artificial *in vitro* conditions may influence the genotype and phenotype of tumour cells, including their potential response to treatment [1–4].

The resistance of cells to anticancer drugs may result from a variety of factors including the ‘stemness state’, epithelial-to-mesenchymal transition (EMT) status and invasion potential, or the expression pattern of genes related to drug metabolism/efflux and cell death defence mechanisms, e.g. the interplay between apoptosis, autophagy and necrosis, mechanisms of DNA damage repair or cell cycle control [5–8].

The aim of the present study was to analyse the most likely mechanisms underlying the phenomenon of glioblastoma resistance by comparing three experimental *in vitro* models of glioblastoma (traditional adherent culture supplemented with serum, serum-free spheroid culture and novel adherent serum-free culture alternative to spheroid system), and to compare the response of these models to treatment with temozolomide (TMZ) or tamoxifen, with regard to cell death type.

Additionally, our analysis of the multifactorial background of glioblastoma drug resistance and chemosensitivity acts as a counterpoint to existing reports which typically recommend individual experimental models for studies of tumour drug response.

## Materials and methods

### Glioblastoma cell culture

Glioblastoma cell cultures were derived from tumour samples obtained from the Department of  Neurosurgery and Oncology of Central Nervous System, Medical University of Lodz, Poland. All procedures (experiments with human tumour-derived cells) were performed in accordance with the ethical standards of the Bioethics Committee of the Medical University of Lodz (reference number of approval RNN/148/08/KE). Glioblastoma cultures were derived from three tumours classified as grade IV according to WHO criteria. Since the tumour samples had been obtained and exploited before the report presenting a current classification of CNS tumour (2016), the genetic status of IDH was not verified and tumours can be classified as *Glioblastoma, NOS* according to the current WHO scheme [9].

The procedure of glioblastoma cell culture generation was optimized on the basis of our previous experience [10,11] and protocols developed by other groups according to an idea of ‘mixed cell culture’ performed without initial separation of derived cells [12].

Tumour tissue was minced in cell culture media and passed through a cell strainer (40 μm; BD Falcon™) to obtain a single cell suspension. Cells were washed with phosphate-buffered saline (PBS) and seeded in T25 cell culture flasks or 6-well plates. The cells were expanded in Dulbecco’s Modified Eagle Medium/Nutrient Mixture F-12 (DMEM/F-12) medium supplemented with 10% fetal bovine serum (FBS; Gibco) and antibiotics (Sigma–Aldrich). Subsequently, the cells were cultured under three different conditions: adherent culture in serum-supplemented medium (DMEM/F12 with 10% FBS), adherent culture in serum-free conditions on commercially available (Corning®Synthemax^™^Surface) vitronectin-mimicking synthetic peptide-acrylate plates (neurobasal medium – NBM with G5, NSC) and spheroid culture in serum-free conditions (NBM medium with N2, B27, epidermal growth factor – EGF, basic fibroblast growth factor – bFGF and heparin). In each case, the culture media were supplemented with GlutaMAX™ (Gibco) and antibiotics (Sigma–Aldrich). Depending on proliferation activity, the cells in adherent cultures were passaged to a new culture dish every 3 to 5 days and expanded for subsequent analyses. The spheroids were grown for 4 to 5 days and passaged using the Accutase treatment.

Further analyses were performed with the use of cells cultured under the particular conditions for at least two to three passages.

The presence of neoplastic cells in the cultures was verified at DNA level (e.g. LOH analyses) and by immunofluorescence detection of appropriate markers of glioblastoma cells (AAAs: IL13Rα, Fra-1) and selected markers of other cell types (endothelial cells: CD34, vWF, CD31; glioblastoma-associated stromal cells, GASCs: α-smooth-muscle actin (α-SMA), FSP) as described previously [11,13–16].

### Immunofluorescence

For immunofluorescence analysis, cell cultures were fixed for 15 min in 4% paraformaldehyde in PBS (and permeabilized with 0.1% Triton X-100 for 10 min, if necessary). Non-specific binding sites were blocked with 2% donkey serum in PBS for 1 h. Subsequently, the cells were incubated for 2 h with the selected primary antibodies (listed below). For visualization, the appropriate species-specific fluorochrome-conjugated secondary antibodies (1:500, donkey anti-rabbit AlexaFluor488; 1:500, donkey anti-mouse Alexa-Fluor594; Molecular Probes) were applied for 1 h in the dark. Controls were created with secondary antibodies alone and matched isotype controls in the place of primary antibodies, and these were processed in the same manner. Slides were mounted with ProLongGold Antifade Reagent with DAPI (Molecular Probes), coverslipped and examined using an Olympus BX-41 fluorescence microscope.

The immunofluorescence assays were performed with the use of the following primary antibodies: anti-IL13 receptor alpha 2 (ab55275, Abcam; WH0003598M1, Sigma–Aldrich), anti-Fra-1 (sc-28310, Santa Cruz Biotech.; PAJ089Hu01, Cloud-Clone Corp), anti-CD44 (sc-7297; Santa Cruz Biotech.), anti-Sry-related HMG box protein 2 (SOX2) (AB5603, Millipore); anti-nestin (sc-23927, Santa Cruz Biotech), anti-N-Cadherin (C3865, Sigma–Aldrich), anti-E-Cadherin (SAB4503751, Sigma–Aldrich), anti-Twist1 (T6451, Sigma–Aldrich) anti-fibronectin (F3648, Sigma–Aldrich); anti-vimentin (V6389, Sigma–Aldrich).

### Invasion assay

The invasion assay was performed using Matrigel invasion chambers (BD Biosciences) according to the manufacturer’s protocol. The glioblastoma-derived cells were cultured for at least three passages under three different conditions: serum-supplemented adherent culture, serum-free adherent culture and serum-free spheroid culture. The cells were then transferred to the top well of the prehydrated Matrigel invasion chambers at a density of 2.5 × 10^4^ cells/well. The number of cells in the spheroids was assessed before the invasion experiment began. The spheroids were generated within 4 days from an initial density of 1 × 10^5^ cells/ml. The cells were then treated with Accutase to obtain a single cell suspension and counted, thus determining the volume of a spheroid culture consisting of the desired amount of 2.5 × 10^4^ cells after 4 days of culture.

The bottom wells of invasion chambers were provided with DMEM/F12 supplemented with 10% FBS as chemoatractant. Each of the upper wells contained one of three media depending on the type of cell culture, the first being DMEM/F12 with 1% FBS, the second NBM medium with G5, and the third being NSC and NBM medium with N2, B27, EGF, bFGF and heparin. The cells were allowed to invade for 24 h. Afterwards, the cells from the top of the chambers were removed and the filters were fixed in 4% paraformaldehyde (Sigma) in PBS for 15 min and subsequently stained fluorescently with DAPI (4,6΄-diaminide-2-phenylindole). Invasion was quantified by counting the number of cells on the underside of the filter from four fields using an Olympus BX-41 fluorescence microscope. The results were expressed as the mean number of cells (means ± SD) from four fields which invaded to the lower surface of the filter from each of the two experiments.

### *MGMT* (O6-methylguanine-DNA methyltransferase) promoter methylation and *MGMT* expression analysis

In order to determine the methylation status of the *MGMT* gene promoter, a modified method of methylation-specific PCR (MSP) based on nested, two-stage PCR was applied. The DNA template was subjected to bisulphite modification. PCR was performed to amplify a 289-bp fragment of the *MGMT* gene, including a part of its CpG-rich promoter. In the first PCR stage, the primers (F: GGA TAT GTT GGG ATA GTT; R: CCA AAA ACC CCA AAC CC) recognized the bisulphite-modified sequence but did not discriminate between methylated and unmethylated alleles. The obtained PCR products were subjected to a stage-2 PCR in which primers specific to a methylated (F: TTT CGA CGT TCG TAG GTT TTC GC; R: GCA CTC TTC CGA AAA CGA AAC G) or unmethylated (F: TTT GTG TTT TGA TGT TTG TAG GTT TTT GT; R: AAC TCC ACA CTC TTC CAA AAA CAA AAC A) template were used. Commercially available positive and negative controls were used (S7822, S7821; Millipore). All assays were performed in duplicate. The PCR products were visualized using agarose gel electrophoresis.

Additionally, the expression of the *MGMT* gene was examined to verify the results of promoter methylation. The relative level of *MGMT* mRNA was measured by real-time PCR using the TaqMan® Gene Expression Assays and KAPA PROBE FAST qPCR Kit Master Mix (2X) Universal (Kapa Biosystems) according to the manufacturer’s protocol. Glyceraldehyde-3-phosphate dehydrogenase (*GAPDH*) was used as a reference gene for normalization of the target gene expression level. The results were analysed using the RotorGene 6000 PCR cycler and software (Qiagen). Normalized relative expression levels of the examined gene were calculated in the tested samples compared with control, as described by Pfaffl et al. [17], based on each sample’s average *C*_T_ value and each gene’s average PCR efficiency. RNA derived from a normal human brain (Total RNA, Brain, Human; Agilent Technologies) was used as a control sample.

### Multi-gene expression analysis – quantitative real-time RT-PCR

The Human Cancer Drug Resistance RT² Profiler™ PCR Array (PAHS-004Z, SABiosciences, Qiagen) was used to profile the expression of 84 genes related to cancer drug resistance and metabolism and involved in the response to chemotherapy.

The total RNA from cells cultured under the particular conditions for at least two or three passages was isolated using the miRNeasy Mini kit (Qiagen) according to the manufacturer’s instructions. cDNA was synthesized from 500 ng of total RNA using the RT2 First Strand Kit (Qiagen).

The samples were analysed using the RT² Profiler PCR Array. Altogether, 84 different genes were amplified simultaneously in the sample. A melting curve analysis was performed to verify that the product consisted of a single amplicon. Real-time PCR was performed using a RotorGene 6000 PCR cycler (Qiagen). Briefly, the reaction mix was prepared from 2× SABiosciences RT2 qPCR Master Mix and 102 μl of sample cDNA. The mixture was added into each well of the PCR Array (20 μl per well). The results were analysed using the RotorGene 6000 software. Normalized relative expression levels of the examined genes in the tested samples versus the control sample were calculated according to Pfaffl et al. [17], based on the mean *C*_T_ value of the sample and the mean PCR efficiency of each gene. RNA derived from a normal human brain (Total RNA, Brain, Human; Agilent Technologies) was used as a control sample.

A change in gene expression of at least 2-fold compared with a control value was considered as up- or down-regulation of the expression of a specific gene, depending on the direction of the change. The results were presented as a heat map generated from the logarithms of obtained values. Genes which presented at least a 2-fold change in expression in comparison with control were selected for further analyses.

The expression of the selected genes was additionally verified with the use of a single primer-assay based on the results of the real-time PCR. Pre-designed commercially available primers were used (Sigma–Aldrich). Reverse transcription was performed using a QuantiTect reverse transcription kit (Qiagen) according to the manufacturer’s protocol. *GAPDH* and  *ACTB* were used as reference genes for normalization of the target gene expression. Each sample was amplified in triplicate in a reaction volume of 10 µl containing 20 ng of cDNA, KAPA SYBR FAST Universal 2× qPCR Master Mix (Kapa Biosystems) and forward and reverse primers. The cycling conditions were set according to the manufacturer’s protocol.

To confirm the specificity of the amplification signal, the gene dissociation curve was considered in each case. Normalized relative expression levels of the examined genes in the tested samples were calculated against a control value according to Pfaffl et al. [17], based on the mean *C*_T_ value of the sample and the mean PCR efficiency of each gene. RNA derived from a normal human brain (total RNA, Brain, Human; Agilent Technologies) was used as a control. The results were expressed as means ± SD.

Additionally, the expression of two genes related to EMT status (*CDH1* and *CDH2*) was examined using quantitative real-time PCR (TaqMan® Gene Expression Assays). The procedure was performed according to the manufacturer’s protocol with the use of the KAPA PROBE FAST qPCR Kit (Kapa Biosystems) and 20 ng of cDNA. The results were calculated according to Pfaffl et al. [17] as described above.

### Drug cytotoxicity assay

The Cell Counting Kit-8 (CCK-8) assay (Sigma–Aldrich) was used to measure the cytotoxicity of tested drugs on glioblastoma cells. The amount of the formazan dye generated by the activity of cellular dehydrogenases is directly proportional to the number of living cells.

Commercially available TMZ (T2577) and tamoxifen (T5648) were purchased from Sigma–Aldrich. The test drug solutions (TMZ, tamoxifen) were prepared in dimethyl sulfoxide (DMSO). A preliminary experiment was performed in order to investigate the influence of  DMSO concentration on the cells. Different concentrations of DMSO, ranging from 0.1% to 5%, were tested. The cells were incubated in the CO_2_-incubator at 37°C for 48 h before assaying for cell viability. The maximum concentration of DMSO which did not decrease cell viability, measured by CCK-8 assay, was found to be 0.5% and this was the maximum permissible concentration used in the following experiments.

Glioblastoma cells cultured under traditional adherent conditions (5 × 10^4^ cells/ml) were grown in 96-well plates for 24 h and treated with selected drugs over a range of concentrations (tamoxifen: 0, 1, 5 and 10 μg/ml; or TMZ: 0, 200, 350 and 500 μM). After 48 h and after 5 days, the extent of cell growth was assessed using the CCK-8 assay. The CCK-8 solution (10 μl) was added to each well, followed by incubation for 3 h at 37°C. The absorbance of the cell culture medium at 450 nm was determined by a multiplate reader (Victor^(TM)^ X; Perkin Elmer). Cell viability was expressed as a percentage of that of the control (untreated) cells. All results in the study were based on at least three experiments.

### Assay for cell apoptosis by annexin V/PI double staining

The extent of apoptosis/necrosis was measured using the annexin V-FITC Apoptosis Detection Kit (Abcam) according to the manufacturer’s instructions. In brief, the glioblastoma cells were plated at a seeding density of 5 × 10^4^ cells/ml under particular culture conditions and treated with different concentrations of tested drugs (tamoxifen: 0, 1, 5 and 10 μg/ml; TMZ: 0, 200, 350 and 500 μM). After 24 h of treatment for tamoxifen or 5 days of treatment for TMZ, the cells were harvested and washed twice with PBS and stained with annexin V-FITC/propidium iodide (PI) at room temperature in the dark for 15 min according to the manufacturer’s instructions.

Data acquisition and analysis were performed by flow cytometry (FACS Canto II; Becton Dickinson). In order to include possible differences between untreated cells derived from the same tumour but growing under different conditions, separate gating criteria were established for the particular cell lines, cultured as three experimental models (G113 10% adh, G113 0% sph, G113 0% adh; G114   10% adh, G114 0% adh; G116 10% adh, G116 0% sph, G116 0% adh). The four populations of cells were analysed. Viable cells were negative for both PI and annexin V; early stage apoptotic cells were positive for annexin V and negative for PI; late stage apoptotic cells were positive for both annexin V and PI and necrotic cells were positive for PI. The results are presented as percentage of apoptotic cells.

### Assay for autophagy detection by FACS with the use of Autophagy Detection Kit

According to the manufacturer (Abcam), the Autophagy Detection Kit (ab139484) measures autophagic vacuoles and monitors autophagic flux in living cells using a novel dye which selectively labels autophagic vacuoles. The cationic amphiphilic tracer (CAT) dye (Green Detection Reagent) has been optimized through the identification of titratable functional moieties which allow for the minimal staining of lysosomes while exhibiting bright fluorescence upon incorporation into pre-autophagosomes, autophagosomes and autolysosomes (autophagolysosomes). The assay offers a rapid and quantitative approach to monitoring autophagy in living cells.

The glioblastoma cells were plated at a seeding density of 5 × 10^4^ cells/ml under particular culture conditions and treated with different concentrations of tested drugs (tamoxifen: 0, 1, 5 and 10 μg/ml; TMZ: 0, 200, 350 and 500 μM). After 24 h of treatment for tamoxifen or 5 days of treatment for TMZ, the cells were harvested and processed according to the manufacturer’s protocol (Abcam).

The control and treated cells were incubated for 30 min at 37°C with Green Detection Reagent and PI to facilitate exclusion of the nonviable cells in the sample during data analysis. Data acquisition and analysis were performed by flow cytometry (FACS Canto II; Becton Dickinson). Finally, the results yielded as mean fluorescence intensity (MFI) were expressed as autophagy activity factor (AAF) calculated according to the equation: 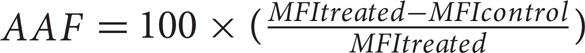.

Fluorescence microscopy images of control and drug-treated cells were performed using an Olympus BX-41 fluorescence microscope to validate the staining capability of Green Detection reagent (autophagy dye).

The analyses were performed as previously described [18].

### Statistical analysis

The results were analysed by nonparametric methods. When more than two groups were analysed, the Kruskal–Wallis test was used to identify any significant difference; if one was found, individual groups were further investigated using the Conover–Inman nonparametric *post hoc* test. In order to compare two groups, the Mann–Whitney U-test was used. In all tests, *P*<0.05 was considered significant.

## Results

### Characteristics of the three types of glioblastoma-derived culture models – AAAs, CSCs markers and EMT status

Glioblastoma cultures were derived from three tumours classified as grade IV (WHO): G113, G114 and G116 tumours. After an initial period of cell culture establishment and expansion, the cells were subjected to three different culture conditions: an adherent culture in traditional serum-supplemented medium (DMEM/F12 medium with 10% FBS), a spheroid culture (NBM medium with N2, B27, EGF, bFGF and heparin) and a novel method of adherent culture on a synthetic vitronectin-mimicking surface in serum-free medium (NBM medium with NSC and G5 supplements). Further experiments were performed on cells growing for at least three passages under different conditions (10% adh, 0% sph, 0% adh).

**Figure 1:**
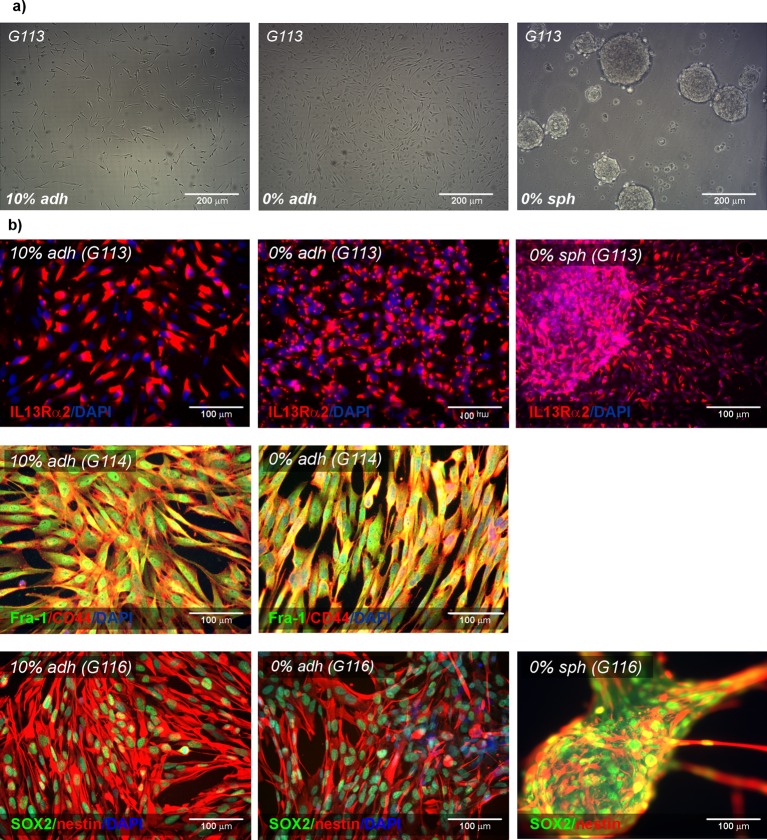
Characteristics of the three types of glioblastoma-derived culture models – AAAs, CSCs markers Sample photomicrographs presenting glioblastoma cells (G113) growing as traditional serum-supplemented adherent culture (10% adh), novel serum-free adherent culture (0% adh) and serum-free spheroid culture (0% sph); (**a**). Representative immunofluorescence results presenting expression of AAAs (IL13Rα2, Fra-1) and the selected CSCs markers (CD44, SOX2, nestin) in glioblastoma cells cultured under three different conditions (**b**).

The G113 and G116 tumours exhibited an ability to grow in all applied models, while the G114 tumour did not generate spheroids. The representative morphology of glioblastoma cells cultured under particular conditions is presented in Figure 1(a).

Under all culture conditions, the tumour-derived cells were found to express IL13Rα2 and Fra-1 (Figure 1). These have been described previously as astrocytoma-associated antigens (AAAs), and have been verified as tools for facilitating the establishment of cell culture and confirming the presence of neoplastic cells in culture [11,13]. In order to detect possible overgrowth by non-tumoral cells, the cultures were monitored for presence of non-tumoral cells (endothelial cells, GASCs) using immunofluorescence detection of appropriate markers (Supplementary Figures S1 and S2). Additionally, the immunofluorescence results were verified by analysis at the DNA level, enabling the detection of anomalies typical for glioblastoma (e.g. LOH analysis) according to the scheme, and methods, described in our previous report [11]. A comparison of immunofluorescence data and genetic analysis results (data not shown) confirmed the neoplastic nature of the cells in all glioblastoma-derived cultures.

**Figure 2:**
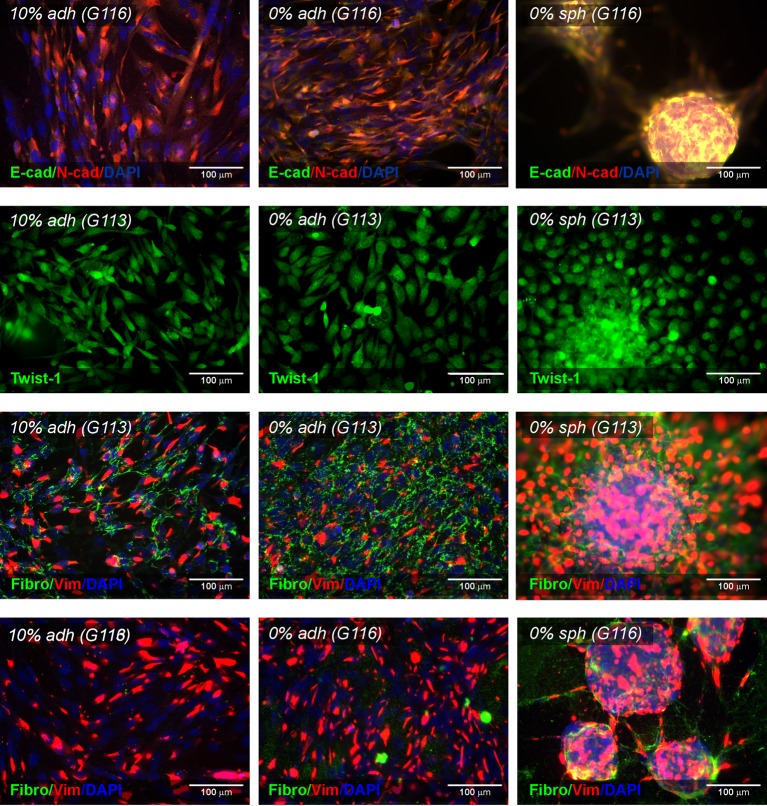
Characteristics of the three types (10% adh, 0% adh, 0% sph) of glioblastoma-derived culture models – EMT status Immunofluorescence photomicrographs showing the expression pattern of EMT-associated protein (E-cadherin, N-cadherin, Twist 1, fibronectin, vimentin) in selected glioblastoma-derived cell cultures (G113 and G116).

The ‘stemness state’ and the EMT status of the cells were then examined: the representative immunofluorescense results for selected tumours are presented in Figures 1 and 2.

The comparative analysis of immunofluorescence results obtained from particular tumours (G113, G114 and G116) cultured as three experimental models (10% adh, 0% sph, 0% adh) revealed the presence of selected CSCs markers (SOX2, nestin and CD44) in G113 and G116 tumour-derived cells. G114 cultures presented modest expression of SOX2 and only individual nestin-positive cells, although CD44 was still strongly expressed. The examined CSC markers were detected in all culture types, although slight variations were observed (Figure 1b).

Similarly, the EMT status of glioblastoma cells was determined by analysing the expression of proteins recognized as EMT transition markers (Figure 2, Supplementary Figure S3). Under all culture conditions, the glioblastoma cells were found to express N-cadherin, but E-cadherin levels were undetectable. These outcomes were confirmed by the real-time PCR results (data not shown).

Additionally, the immunocytochemical results revealed the expression of other markers of the EMT transition/mesenchymal state: Twist1, vimentin and fibronectin. All examined tumours demonstrated Twist1 expression in adherent culture and spheroids, with the nuclear signal being detected in G113 and G114 cells but not in G116, where only the cytoplasmic expression pattern was observed. Vimentin was expressed in all cases, with subtle variations observed between culture conditions. Fibronectin was detected at a moderate level in all but the G116-derived cells, which appeared to be fibronectin-negative in adherent cultures but fibronectin-positive in the case of spheroids.

Our results demonstrate that the use of a Synthemax xeno-free surface enables the propagation of glioblastoma-derived cells as a monolayer culture without serum supplementation, and the cells were positive for the examined AAAs (IL13Rα2, Fra-1), the analysed CSCs markers and the selected markers of EMT (Figures 1 and 2).

### Invasion ability of glioblastoma cells cultured under different conditions

The invasion capability of glioblastoma cells derived from the G113, G116 and G114 tumours cultured under three different conditions were examined using the Matrigel invasion chamber system.

**Figure 3:**
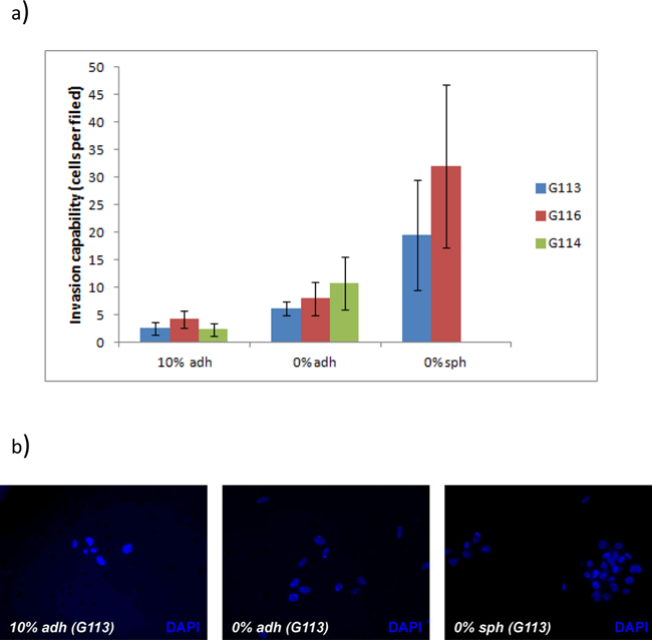
The invasion ability of glioblastoma cells cultured under three different conditions *in vitro* (**a**) Quantitative data demonstrated significant differences (*P*<0.05) in the invasion ability of glioblastoma cells grown in serum-supplemented adherent culture (10% adh), serum-free adherent culture (0% adh) and serum-free spheroid culture (0% sph). (**b**) Sample microphotographs of tumour cells which invaded through the Matrigel-coated filters and were fluorescently stained with DAPI.

For all tested tumours, comparative analysis showed a significant difference between the number of cells invading through the Matrigel from spheroids, adherent cells cultured in serum-free medium and adherent cells cultured in serum (*P*<0.05). The highest invasion capability was presented by the cells cultured as spheroids, while the cells growing under standard serum conditions demonstrated the lowest invasion potential (Figure 3a). The results were visualized as fluorescent microphotographs of the lower surface of the filters (Figure 3b). The results confirm that glioblastoma cells cultured as monolayers on a Synthemax surface in serum-free conditions retain their invasion capability *in vitro*.

### The expression profile of genes related to tumour drug-resistance in glioblastomas cultured in three distinct models (a multi-gene expression analysis)

**Figure 4:**
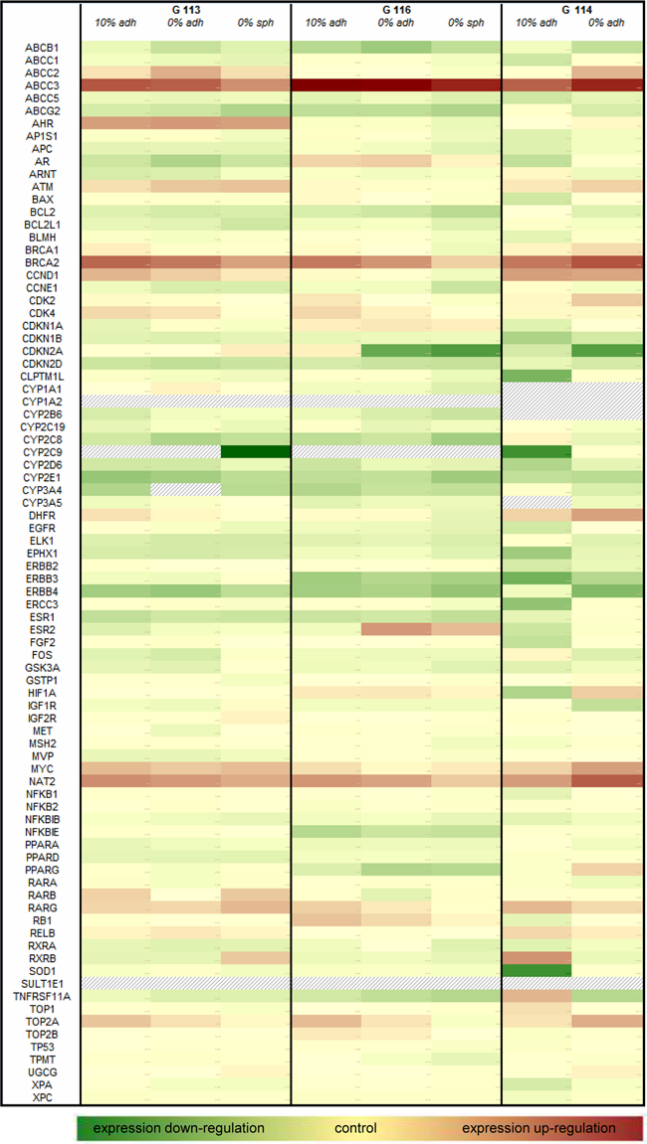
A heat map showing the expression profile of 84 genes included in the Human Cancer Drug Resistance RT² Profiler™ PCR Array in glioblastoma cells cultured under three different conditions in comparison with control (normal human brain) The relative expression for control is assumed to be 1; the examined samples presented up-regulation of tested genes (values above 1) or down-regulation of tested genes (values below 1). The fields filled with grey strips indicate a non-detectable expression level. Logarithmic values of the data were used for heat map generation.

The results of the real-time PCR array for the panel of genes related to the phenomenon of cancer drug resistance are shown as a heat map presenting the scale of fold change in examined gene expression normalized to that of a normal control brain sample (Figure 4).

The expression of genes presenting at least a 2-fold change (up-regulation) in comparison with the control value was additionally verified by means of a single-primer real-time PCR assay. Based on this criterion, 17 genes were selected for further analysis. The obtained results allowed for identification of genes differentially expressed in glioblastoma cells derived from particular culture models.

Comparing the particular cell culture conditions (adherent culture in 10% FBS, serum-free adherent culture and spheroid culture of G113 and G116 tumours), a gradually decreasing trend was observed regarding the expression of ten genes, although this was insignificant in some cases. The G114-derived culture, devoid of its ability to form spheres, demonstrated higher levels of examined gene expression in adherent serum-free culture than in the serum-supplemented culture. This expression pattern concerned mainly the genes involved in drug resistance and metabolism, DNA damage and repair and cell cycle control: ATP-binding cassette sub-family C (CFTR/MRP), member 3 (*ABCC3*); topoisomerase 2 alpha (*TOP2A*); dihydrofolate reductase (*DHFR*); N-acetylotransferase 2 (*NAT2*); breast cancer suppressor gene 1 (*BRCA1*); breast cancer suppressor gene 2 (*BRCA2*); cyclin D1 (*CCND1*); cyclin-dependent kinase 2 (*CDK2*); cyclin-dependent kinase 4 (*CDK4*).

The expression of genes encoding hormone receptors and transcription factors did not present such a consistent pattern. The detailed results of the comparative analyses are listed in Table 1.

**Table 1:** Expression pattern of genes related to cancer drug-resistance in different types of glioblastoma-derived cultures

Functional gene grouping	Gene	G113				G116				G114		
		10% adh (A)	0% adh (B)	0% sph (C)	*P*<0.05 (*)	10% adh (D)	0% adh (E)	0% sph (F)	*P*<0.05 (*)	10% adh (G)	0% adh (H)	*P*<0.05 (*)
Drug resistance	***ABCC2***	2.42 ± 0.19	7.08 ± 0.36	2.56 ± 0.36	*A* versus *B*	0.94 ± 0.22	0.99 ± 0.09	0.43 ± 0.04	*E* versus *F*	0.76 ± 0.16	6.30 ± 1.21	*G* versus *H*
					*B* versus *C*				*D* versus *F*			
	***ABCC3***	45.53 ± 7.15	37.93 ± 8.15	13.67 ± 0.82	*B* versus *C*	374.32±19.99	309.18 ± 69.28	132.67 ± 32.18	*E* versus *F*	35.96 ± 4.62	131.52 ± 17.09	*G* versus *H*
					*A* versus *C*				*D* versus *F*			
	***TOP2A***	4.51 ± 0.69	2.51 ± 0.29	1.51 ± 0.31	*A* versus *B*	5.46 ± 1.06	2.32 ± 1.03	0.40 ± 0.14	*D* versus *E*	2.34 ± 1.13	7.32 ± 3.08	*G* versus *H*
					*B* versus *C*				*E* versus *F*			
					*A* versus *C*				*D* versus *F*			
Drug metabolism	***DHFR***	2.51 ± 0.21	1.58 ± 0.25	0.77 ± 0.04	*A* versus *B*	1.44 ± 0.28	1.42 ± 0.43	0.19 ± 0.07	*E* versus *F*	3.31 ± 1.73	9.69 ± 0.46	*G* versus *H*
					*B* versus *C*				*D* versus *F*			
					*A* versus *C*							
	***NAT2***	14.69 ± 3.31	11.28 ± 2.15	7.61 ± 2.05	*A* versus *C*	12.28 ± 0.61	9.25 ± 0.58	4.02 ± 0.27	*D* versus *E*	10.49 ± 1.67	39.41 ± 6.08	*G* versus *H*
									*E* versus *F*			
									*D* versus *F*			
DNA damage and repair	***ATM***	2.56 ± 0.74	4.27 ± 0.93	4.77 ± 0.53	*A* versus *C*	1.50 ± 0.28	1.39 ± 0.13	1.30 ± 0.39	n.d.	2.16 ± 0.16	3.46 ± 0.40	*G* versus *H*
	***BRCA1***	1.91 ± 0.52	1.37 ± 0.41	0.63 ± 0.17	*B* versus *C*	1.47 ± 0.38	0.9 ± 0.24	0.26 ± 0.05	*E* versus *F*	1.64 ± 0.44	2.73 ± 0.82	*G* versus *H*
					*A* versus *C*				*D* versus *F*			
	***BRCA2***	32.54 ± 2.68	22.94 ± 3.09	9.67 ± 0.60	*A* versus *B*	22.01 ± 3.83	12.38 ± 1.53	3.61 ± 0.08	*D* versus *E*	22.69 ± 2.14	47.64 ± 5.83	*G* versus *H*
					*B* versus *C*				*E* versus *F*			
					*A* versus *C*				*D* versus *F*			
Cell cycle	***CCND1***	6.19 ± 1.16	3.71 ± 0.43	2.12 ± 0.68	*A* versus *B*	1.46 ± 0.19	1.16 ± 0.45	0.29 ± 0.11	*E* versus *F*	10.20 ± 2.11	8.88 ± 0.15	n.d.
					*B* versus *C*				*D* versus *F*			
					*A* versus *C*							
	***CDK2***	1.46 ± 0.18	1.41 ± 0.21	0.89 ± 0.24	*B* versus *C*	2.22 ± 0.28	1.00 ± 0.24	0.44 ± 0.08	*D* versus *E*	1.56 ± 0.07	4.03 ± 0.71	*G* versus *H*
					*A* versus *C*				*E* versus *F*			
									*D* versus *F*			
	***CDK4***	2.98 ± 0.26	2.42 ± 0.73	1.19 ± 0.10	*B* versus *C*	2.99 ± 0.48	1.77 ± 0.06	1.13 ± 0.28	*D* versus *E*	1.54 ± 0.10	1.55 ± 0.29	n.d.
					*A* versus *C*				*E* versus *F*			
									*D* versus *F*			
Hormone receptors	***AR***	0.05 ± 0.00	0.02 ± 0.01	0.05 ± 0.02	*A* versus *B*	3.26 ± 0.30	3.75 ± 0.81	1.74 ± 0.43	*E* versus *F*	0.04 ± 0.01	1.65 ± 0.45	*G* versus *H*
					*B* versus *C*				*D* versus *F*			
	***ESR2***	0.13 ± 0.02	0.34 ± 0.01	0.41 ± 0.17	*A* versus *B*	0.29 ± 0.06	11.52 ± 3.08	5.39 ± 0.96	*D* versus *E*	0.06 ± 0.02	0.53 ± 0.22	*G* versus *H*
					*A* versus *C*				*E* versus *F*			
									*D* versus *F*			
	***RARB***	3.46 ± 0.18	0.95 ± 0.18	4.21 ± 0.51	*A* versus *B*	1.29 ± 0.19	0.16 ± 0.03	1.38 ± 0.11	*D* versus *E*	1.20 ± 0.13	0.63 ± 0.27	*G* versus *H*
					*B* versus *C*				*E* versus *F*			
					*A* versus *C*							
	***RARG***	3.14 ± 0.47	3.05 ± 0.44	5.84 ± 0.25	*B* versus *C*	3.65 ± 0.53	2.10 ± 0.24	1.32 ± 0.15	*D* versus *E*	5.80 ± 0.34	2.90 ± 0.37	*G* versus *H*
					*A* versus *C*				*E* versus *F*			
									*D* versus *F*			
Transcription factors	***AHR***	10.15 ± 1.82	11.28 ± 3.03	9.89 ± 1.31	n.d.	0.45 ± 0.10	0.44 ± 0.12	0.23 ± 0.09	*D* versus *F*	1.05 ± 0.08	1.52 ± 0.56	n.d.
	***MYC***	5.65 ± 0.38	4.09 ± 0.67	5.36 ± 1.14	*A* versus *B*	2.57 ± 0.43	1.55 ± 0.43	1.97 ± 0.39	*D* versus *E*	3.38 ± 0.10	10.12 ± 0.39	*G* versus *H*

Results of quantitative real-time PCR showing fold-changes in gene expression levels in comparison with control (normal human brain). Comparative analyses of gene expression levels between particular culture conditions for each tumour revealed statistical significance for the most of the detected differences (*P*<0.05); the values marked with (*).

10% adh - adherent serum-supplemented culture; 0% adh - adherent serum-free culture; 0% sph - serum-free spheroid culture; n.d. – no differences

### Initial analyses of cytotoxic effect of tamoxifen and temozolomide on glioblastoma cells

**Figure 5:**
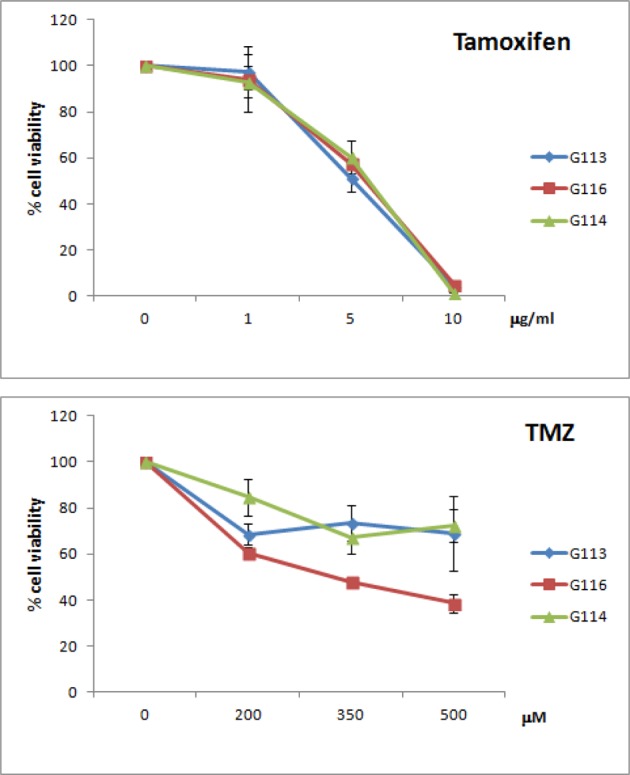
Dose-dependent chemotherapy response of glioblastoma cells to tamoxifen and TMZ treatment under traditional culture conditions Cell viability was assessed by the CCK-8 assay. Toxic effects were detected after 2 and 5 days of tamoxifen and TMZ treatment respectively. G113 and G114 presented *MGMT* gene expression, while G116 is MGMT-negative.

Due to the heterogeneity of glioblastoma, an initial protocol of treatment was applied in order to assess the time- and dose-dependent cytotoxicity for a particular tumour-derived culture under standard culture conditions. The potential response/resistance of G113, G114 and G116 glioblastoma cells was tested with the use of two known therapeutics: tamoxifen and TMZ. The cells were exposed to increasing doses of tested drugs (tamoxifen: 0, 1, 5 and 10 μg/ml; TMZ: 0, 200, 350 and 500 μM). The range of doses was selected based on previously published data [19,20]. As the preliminary experiment showed that DMSO had a visible negative effect on cell viability at concentrations above 0.5%, the solvent concentration in final experiments did not exceed 0.5%. Cell viability was assessed with the CCK-8 assay 2 days after the addition of tamoxifen, and 5 days after that of TMZ. The toxic effects of tamoxifen were observable after 2 days and TMZ after 5 days of treatment (Figure 5).

The obtained results allowed for doses and time of treatment for further experiments based on FACS analyses to be selected. Further experiments were aimed at finding potential differences between the three tumour cell cultures in response to the tested drugs with recognized cytotoxicity against glioblastoma. The most important criteria in the selection of these drugs included their known application in routine glioblastoma treatment or clinical trials, and the potential to induce both apoptosis and autophagy. Additionally, tamoxifen appeared to be effective in the case of TMZ-resistant tumours.

### Analyses of cell death types/pathways induced by tamoxifen and temozolomide in three models of glioblastoma culture

The second stage of the cytotoxicity experiments was based on an analysis of mechanisms of death/resistance related to apoptosis, necrosis and autophagy. In order to optimize the time to monitor not only the apoptotic but also the autophagy status, the effects of TMZ were examined after 5 days of drug exposure and tamoxifen after 24 h.

**Figure 6:**
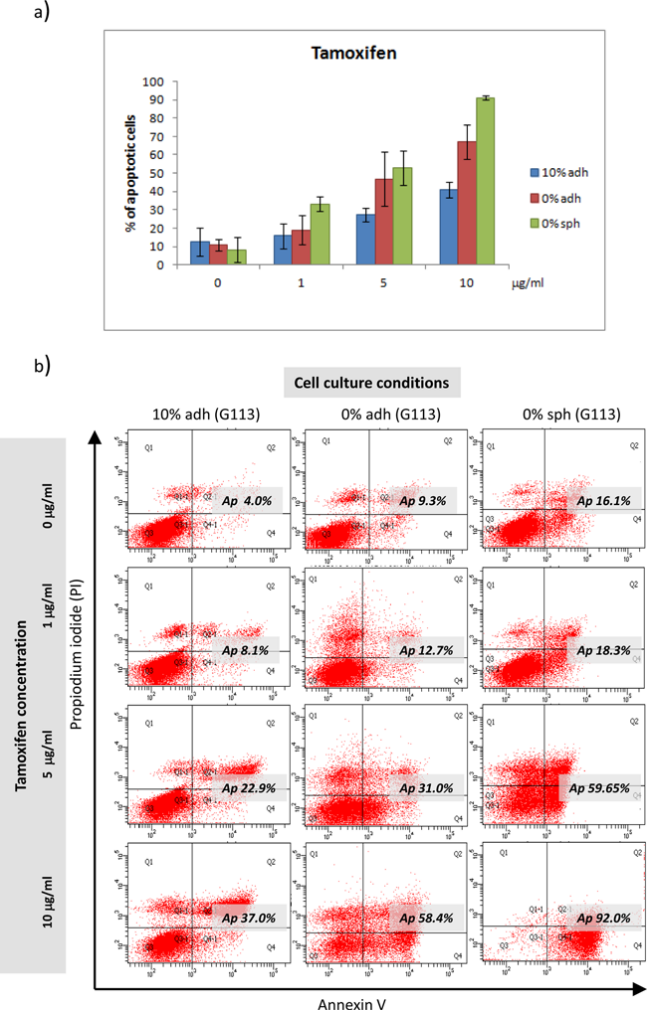
Apoptotic response in three different types of culture of glioblastoma cells treated with tamoxifen (**a**) Quantitative analysis revealed differences in the apoptotic response of glioblastoma cells cultured in different conditions observable at higher drug concentrations. Each bar represents the mean percentage (±SD) of pooled data of apoptotic cells yielded from all three tumours (G113, G114 and G116) cultured under the same conditions (10% adh, 0% adh, 0% sph). (**b**) Sample results (the G113 culture) of the FACS analysis demonstrating the extent of apoptosis/necrosis measured by annexin V-FITC/PI staining for control cells and three concentrations of drug under three culture conditions.

The FACS analyses based on annexin/PI staining allowed for the evaluation of the percentages of apoptotic, necrotic and viable cells after the tamoxifen and TMZ treatment applied in three culture models: the serum-supplemented adherent culture (10% adh), the serum-free adherent culture (0% adh) and the serum-free spheroid culture (0% sph). In the case of the tamoxifen treatment, the results demonstrated different chemosensitivity of glioblastoma cells depending on the cell culture model. The compilation of results obtained for the G113, G114 and G116 tumours following tamoxifen treatment is given in Figure 6(a). The comparative analysis revealed significant differences in tamoxifen sensitivity, visible as a higher percentage of apoptotic cells in adherent and spheroid serum-free cultures (*P*<0.05) at drug concentrations of 5 and 10 μg/ml. The percentage of necrotic cells did not exceed 20% in any analysed population. The sample results (G113) are presented in Figure 6(b).

**Figure 7:**
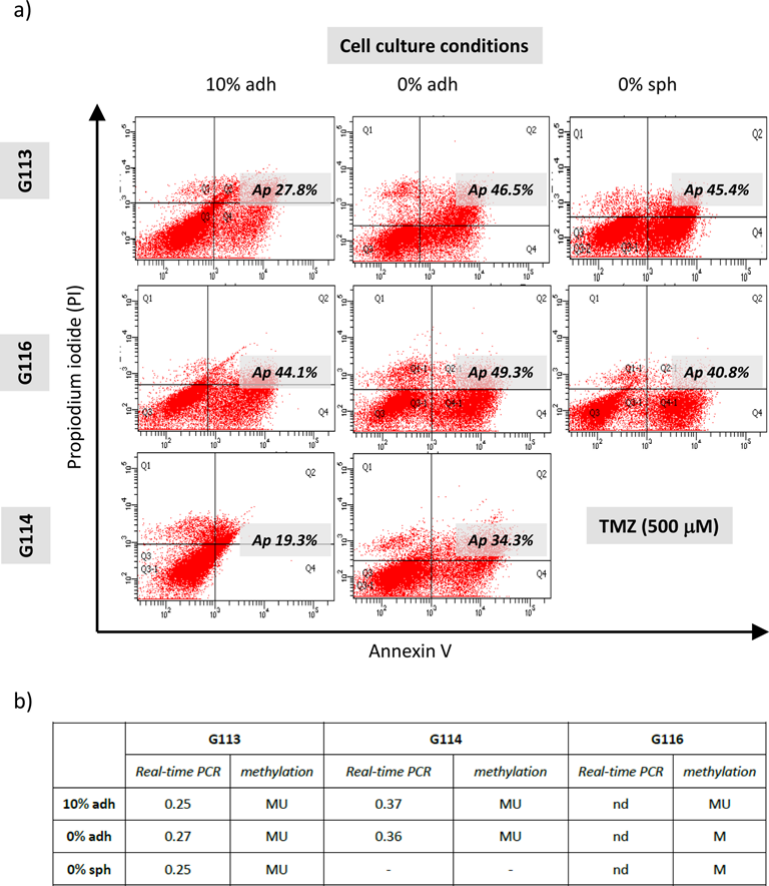
*MGMT-*status dependent apoptotic response in three different types of culture of glioblastoma cells treated with TMZ (**a**) The representative results of FACS analysis demonstrating the extent of apoptosis/necrosis measured by annexin V-FITC/PI staining in control cells and for the highest applied concentration of TMZ under three culture conditions for particular tumours (G113, G114 and G116). The differences in sensitivity to TMZ were visible mainly at the highest dose of TMZ in the case of the MGMT-positive G113 and G114 cultures, reflected as higher percentage of apoptotic cells under serum-free conditions. No such difference was observed in the case of the G116 culture characterized by the lack of *MGMT* expression. In order to include possible differences between untreated cells derived from the same tumour but growing under different conditions, separate gating criteria were established for the particular cell lines, cultured as three experimental models. (**b**) Analysis of the *MGMT* status in G113 and G114 cases showed no differences in gene promoter methylation or expression for particular type of cell culture. The table summarizes the results of real-time PCR presented as the relative expression levels of *MGMT* in comparison with control – normal human brain (nd – non-detectable level) and methylation status of the *MGMT* promoter (MU – methylated/unmethylated; M – methylated).

The results obtained after TMZ treatment were analysed separately due to differences in *MGMT* expression status and promoter methylation in particular tumours (results in Figure 7).

The results of FACS analyses performed for three types of cultures revealed differences in sensitivity to TMZ visible at the highest drug concentration (500 μM) manifesting as higher percentages of apoptotic cells under the serum-free conditions, in the case of the G113 and G114 culture. No such difference was observed in the case of the G116 culture characterized by a lack of *MGMT* expression (Figure 7). The analysis of the *MGMT* status showed no differences in gene promoter methylation or expression for any particular cell culture type (Figure 7b).

Additionally, the autophagy statuses were monitored in glioblastoma culture after drug treatment. The analysis was performed by FACS testing and verified by fluorescence microscopy as previously described [18].

In brief, the autophagy intensity was expressed as AAF, a parameter assessed on the basis of MFI detected by FACS, and reflected the quantity of pre-autophagosomes, autophagosomes, and autolysosomes (autophagolysosomes) present in an analysed cell population under particular treatment conditions and control cell populations.

**Figure 8:**
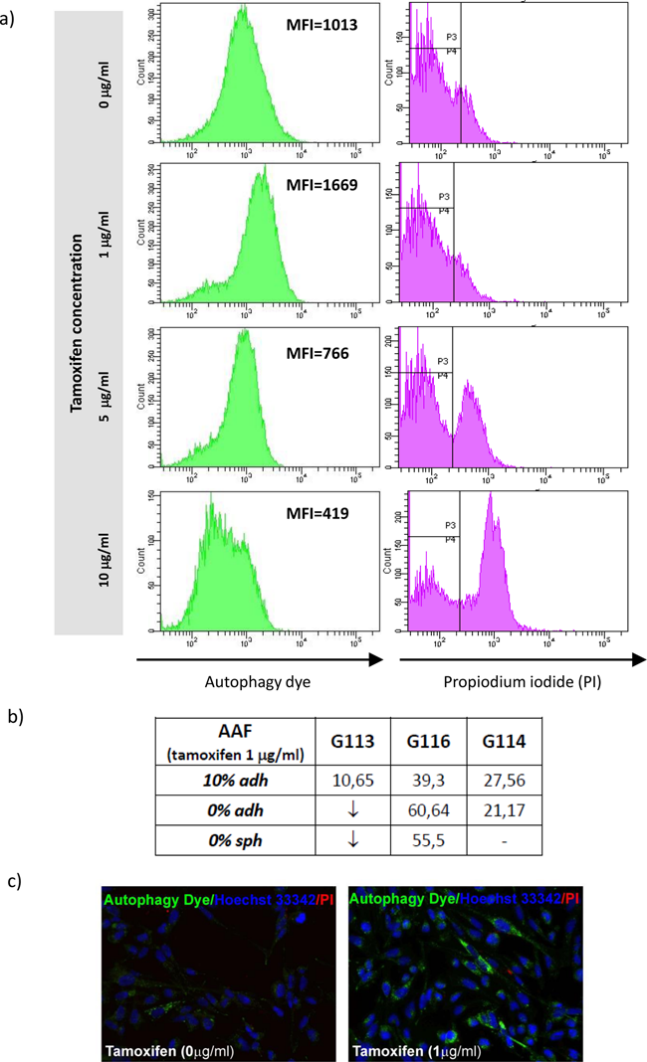
Autophagy status in glioblastoma cells subjected to tamoxifen treatment Results of FACS analysis demonstrated intensification of autophagy process (showed as mean fluorescence intensity – MFI) only at the lowest drug concentration. Application of higher drug doses resulted in silencing of this process and increasing percentage of necrotic cells detected by means of the PI accumulation. (**a**) The sample results of FACS analysis (G116 culture, 10% adh) based on autophagic vacuoles labelling and PI staining for control cells and the cells treated with increasing tamoxifen concentration. (**b**) The results reflecting autophagy intensity following the administration of the lowest dose of tamoxifen in particular glioblastoma cultures. Data are expressed as AAF calculated according to the equation: 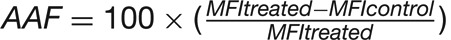. ↓ - the intensity of autophagy below the level detected in control. (**c**) Fluorescence microscopy images of control and drug-treated cells confirming the staining capability of autophagy dye (green detection reagent) used for FACS analysis.

The analysis performed after tamoxifen treatment showed that the autophagy process is being intensified only at the lowest drug concentration (1 μg/ml). At higher concentrations, the detected MFI values were lower than those of the control cells (Figure 8). The observed decrease in autophagy intensity was accompanied with the increase in PI accumulation (Figure 8) indicating an intensification of the apoptosis and necrosis processes described earlier (Figure 6).

**Figure 9:**
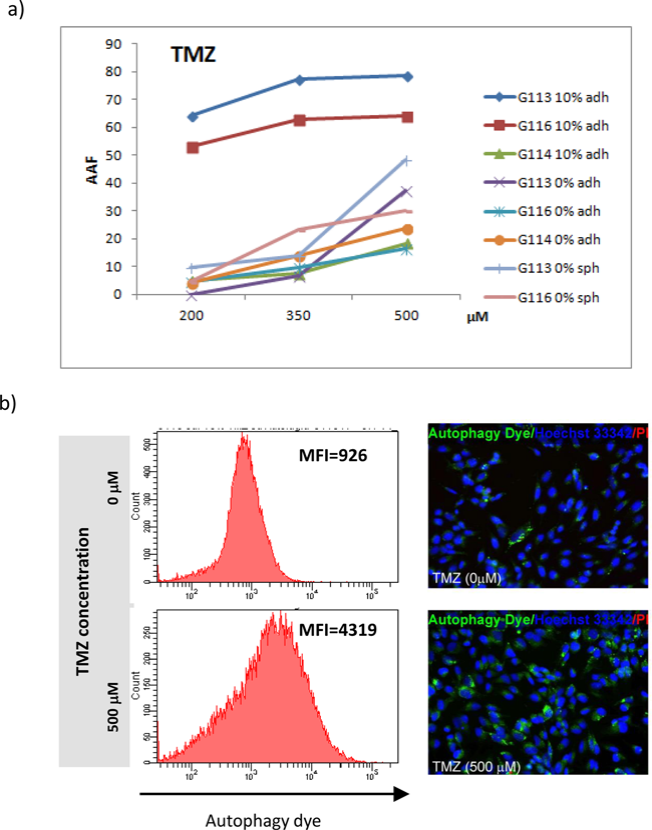
Autophagic response in glioblastoma cells treated with increasing concentration of TMZ (**a**) Quantitative analysis showed dose-dependent intensification of autophagy process following the TMZ application and revealed differences in autophagy intensity between culture conditions detected for G113 and G116 tumours. Data are expressed as AAF calculated according to the equation: 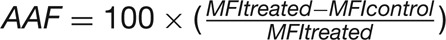. (**b**) The sample results of FACS analysis (G113 culture) based on autophagic vacuoles labelling in control cells and cells treated with the highest TMZ concentration confirmed by fluorescence microscopy images of control and drug-treated cells (MFI - mean fluorescence intensity).

The TMZ treatment resulted in a gradual increase in AAF under particular drug concentrations (Figure 9).

Additionally, in the case of the G113- and G116-derived populations, the autophagy process following the drug treatment was much more intensified in serum cultures when compared with the serum-free ones. No such divergence was observed in the case of the G114 tumour (Figure 9). The TMZ treatment resulted in both autophagy and apoptotic processes in the analysed cell population; however, the highest applied concentration (500 μM) yielded a maximum of 40–50% of apoptotic cells, depending on the cell culture type. In all analysed populations, the percentage of necrotic cells did not exceed 10% (Figure 7).

## Discussion

A number of recent works discuss the problem of choosing the best experimental model for tumour cell culture. Several studies indicate spheroid culture as the experimental model most relevant for studying the biology of the tumour and its response to treatment; however, detailed analyses seem to also present arguments for adherent culture. Apart from the technical opportunities related to spheroid models, the molecular and phenotypic heterogeneity of glioblastoma may be manifested as the incapability of particular primary tumour cultures to form spheroids [1,2,21,22].

Our work demonstrates the difference in therapy resistance capacity presented by glioblastoma cells cultured under three different conditions: traditional adherent culture with serum, serum-free spheroid culture and novel serum-free adherent culture using a modified vitronectin-mimicking surface as an alternative method to the spheroid system. The study comprised results yielded with the use of two glioblastoma-derived cultures (G113 and G116) able to grow under all three conditions and one glioblastoma culture (G114) without a spheroid formation capacity.

Tumour resistance is a complex phenomenon may be easily affected by artificial *in vitro* conditions. Our study compares a panel of selected features potentially underlying the phenomenon of tumour resistance, possibly influenced by *in vitro* environmental factors.

Initially, the glioblastoma-derived cells were characterized with the use of IL13Rα2 and Fra-1 markers; consistent results were observed for all tested tumours and no detectable/obvious differences were revealed by AAA staining.

The stemness status is indicated as the key factor deciding the potential response to treatment [5,7]. Our results revealed the expression of selected CSCs markers in all tested tumour-derived cultures, with subtle variations in expression level being detectable between different culture conditions; the G113- and G116-derived cells, possessing the ability to grow in spheroids, were positive for all tested markers in contrast with the G114 adherent cells.

The EMT is also believed to be a factor involved in tumour resistance capacity [7,23]. Our models of glioblastoma cultures were characterized according to several markers of EMT status [24]; immunocytochemistry analysis confirmed the expression of four of the selected EMT status markers (N-cadherin, fibronectin, vimentin and Twist-1) in the examined tumours, although variations were detected between culture conditions. The immunocytochemistry results indicated that the spheroids demonstrated strong expression of all tested markers. This observation seems to be consistent with our invasion assay data indicating the spheroids are the most invasive culture type.

Our further results based on multi-gene expression analyses related to cancer drug resistance allowed for the identification of 17 genes which are overexpressed in glioblastoma cells. The selected genes belong to different functional groups including drug efflux and metabolism, DNA damage and repair, cell cycle regulation, hormone receptors and transcription factors. The comparative analyses also revealed differences in the expression levels of tested genes between particular culture conditions.

The detected expression pattern of genes related to drug resistance and metabolism, DNA damage and repair and cell cycle regulation (*ABCC3*,* TOP2A*,* DHFR*,* NAT2*,* BRCA1*,* BRCA2*,* CCND1*,* CDK2* and* CDK4*) seems to be in line with our results obtained in the cytotoxicity analyses for G113 and G116 tumours presenting enhanced drug resistance in adherent serum-supplemented culture. Among these genes, the highest levels of transcripts were detected for *ABCC3, NAT2* and *BRCA2* (Table 1). The detected group of overexpressed genes includes some potential therapeutic targets (*ABCC3, CCND1, CDK2* and *CDK4*) [25,26]. Additionally, previous investigations have revealed a mutual interrelation between some of these genes (*TOP2A, BRCA1, BRC*A2 and*ABCC3*), as well as the effectiveness of the agents used in our cytotoxicity study [27–29]. Another interesting observation is the elevated expression of *NAT2* detected in our glioblastoma culture: since this overexpression has not yet been reported for glioblastomas, it may prove to be a potential new therapeutic target.

An analysis of the literature found cancer drug resistance to be a complex phenomenon depending on a variety of interrelated factors. A review by Singh et al. [7] highlighted a possible relationship between EMT transition and CSC status, two factors favouring the drug resistance of cancer cells. Johannessen et al. [30] regarded the mechanisms of DNA repair in the context of CSCs. Recent reports regarding the relationship between the EMT phenomenon and drug resistance have emphasized the link between EMT status and overexpression of genes related to drug efflux and DNA repair [23,31]. Such a complex background of cancer drug resistance makes it difficult to find a simple and unequivocal linkage between the revealed expression profile of examined genes and chemosensitivity of glioblastoma cultures tested in our study.

The cytotoxicity experiments were performed with the use of two known therapeutics: tamoxifen and TMZ. The differences in response of particular culture models to tamoxifen treatment were clearly observable at the highest drug concentration, indicating that spheroid culture was the most sensitive model. The adherent serum-free cultured glioblastoma cells were also more sensitive than the traditional culture supplemented with serum.

The analysis of glioblastoma chemosensitivity to TMZ was preceded by an analysis of *MGMT* promoter methylation and gene expression status, as these two factors have been found to determine treatment efficacy [5,6,32]. As expected, MGMT-negative G116 demonstrated the highest sensitivity to TMZ treatment, regardless of the cell culture conditions. The MGMT-positive G113 and G114 tumours presented different levels of drug resistance depending on the culture conditions, despite no differences in the expression of *MGMT* or its promoter methylation. However, differences were noticed in the expression of the *BRCA1* and *BRCA2* genes, which are related to mechanisms of DNA repair. Recent investigations revealed an association between TMZ resistance and the level of expression of these proteins in glioma [28]. At the highest drug concentration used in the present study, the chemosensitivity of G113 populations cultured under both serum-free conditions was similar to the response of the MGMT-negative G116 cells. In the case of the *MGMT*-expressing tumours (G113 and G114), the serum conditions resulted in decreased efficacy of the TMZ treatment. Additionally, our comparative analyses showed that tamoxifen appeared to be an effective cytotoxic agent against glioblastoma cells resistant to TMZ, which is consistent with previous report [33].

Since both TMZ and tamoxifen are considered as autophagy inducers, the autophagy process was monitored in parallel to the analyses of apoptosis and necrosis.

Several studies have indicated that the toxic effect of tamoxifen on cancer cells was exerted via oestrogen receptor-dependent pathways. Nevertheless, recent papers have also noted that tamoxifen has toxic effects on ER-negative malignancies. He et al. [33] and Hui et al. [34] demonstrated that glioma cell lines lacking oestrogen receptors have an apoptotic response to tamoxifen treatment. In line with previous investigations, our real-time PCR results reveal no *ESR1* expression (ERα; oestrogen receptors type-1) in any of the tested cases, but higher *ESR2* expression (ERβ; oestrogen receptors type-2) in G116-derived cells cultured under serum-free conditions only. Therefore, it cannot be excluded that tamoxifen may act via an ERβ-dependent pathway, in a manner similar to the mechanism described for ERα-negative breast cancer [35].

Despite the fact that tamoxifen is a known autophagy inducer, our findings demonstrate that tamoxifen treatment resulted in only a periodical increase in autophagy, detected at the lowest drug concentration, with the apoptotic and necrotic populations in the minority. However, increasing tamoxifen concentrations yielded a higher percentage of apoptotic cells.

A distinct effect was observed following the TMZ treatment: increasing drug concentration was associated with enhancement of the autophagy accompanying the apoptotic process. Additionally, the serum-cultured G113 and G116 cells presented higher levels of autophagy in comparison with populations cultured under both serum-free conditions, irrespective of the drug concentrations. Such differences were not observed in the G114 tumour.

Autophagy is regarded as both a pro-survival or pro-death process, depending on the cellular context. Previous investigations employing the glioblastoma models presented inconsistent results indicating TMZ as apoptosis or autophagy inducer [8,36,37]. Our results demonstrated that apoptosis and autophagy are parallel processes, both of which occurred following the TMZ treatment. Knizhnik et al. [37] suggested that TMZ-induced autophagy serves as a survival mechanism inhibiting apoptosis. Their results seem to be consistent with our outcomes obtained under serum conditions for *MGMT*-expressing tumours: apoptosis and autophagy were detected as parallel processes in glioblastoma cells cultured under serum-free conditions, which is contrary to the findings of a previous study by Knizhnik et al. [37] in the presence of serum. The discussed discrepancies in the effects of TMZ treatment may result from differences in culture conditions; these are reflected in our findings, indicating changes in the intensification of autophagy and apoptosis processes in different culture models.

## Conclusions

Our comparative cytotoxicity analysis indicated traditional adherent culture as being more resistant than serum-free adherent and spheroid culture, which present the greatest sensitivity. Although it is not surprising that the artificial *in vitro* environment can modify the cellular phenotype and drug-responsiveness, an important finding is that the extrinsic factors can change not only the molecular background of drug resistance and treatment efficacy, but also the mechanisms or pathways of cell death.

Additionally, the characteristics of glioblastoma-derived cells demonstrate that our novel method of adherent culture can be considered as an alternative serum-free system for tumours without spheroid formation capacity. However, our results suggest that parallel exploitation of different experimental models, i.e. changes in *in vitro* tumour environment, can unveil the spectrum of cancer cell resistance capability and reveal potential therapeutic targets, especially in relation to intra-heterogeneous glioblastomas.

## Abbreviations

α-SMA: α-smooth-muscle actin
0% adh: serum-free adherent culture
0% sph: serum-free spheroid culture
10% adh: serum-supplemented adherent culture
AAA: astrocytoma-associated antigen
AAF: autophagy activity factor
ABCC3: ATP-binding cassette sub-family C (CFTR/MRP), member 3
AR: androgen receptor
bFGF: basic fibroblast growth factor
BRCA1: breast cancer suppressor gene 1
BRCA2: breast cancer suppressor gene 2
CCK-8: cell counting kit-8
CCND1: cyclin D1
CD31: (PECAM1) platelet and endothelial cell adhesion molecule 1
CD34: CD34 antigen
CD44: cluster of differentiation antigen-44
CDK2: cyclin-dependent kinase 2
CDK4: cyclin-dependent kinase 4
CSC: cancer stem cell
DAPI: 4,6΄-diaminide-2-phenylindole
DHFR: dihydrofolate reductase
DMSO: dimethyl sulfoxide
DMEM/F-12: Dulbecco’s Modified Eagle Medium/Nutrient Mixture F-12
EGF: epidermal growth factor
EMT: epithelial-to-mesenchymal transition
EphA2: Ephrin Type-A Receptor 2
ESR1 (ERα): oestrogen receptors type-1 (type α)
ESR2 (ERβ): oestrogen receptors type-2 (type β)
FBS: fetal bovine serum
Fra-1: Fos-related antigen 1
FSP: fibroblast surface protein
GAPDH: glyceraldehyde-3-phosphate dehydrogenase
GASCs: glioblastoma associated stromal cells
IL13Rα2: IL13 receptor alpha 2
MFI: mean fluorescence intensity
MGMT: O6-methylguanine-DNA methyltransferase
MPNSTs: malignant peripheral nerve sheath tumours
MSP: methylation-specific PCR
NAT: N-acetyltransferase
NAT2: N-acetylotransferase 2
NBM: neurobasal medium
PBS: phosphate-buffered saline
PI: propidium iodide
SOX2: Sry-related HMG box protein 2
TMZ: temozolomide
TOP2A: topoisomerase 2 alpha
Twist1: Twist-related protein 1
vWF: von Willebrand factor
